# Immediate early genes in social insects: a tool to identify brain regions involved in complex behaviors and molecular processes underlying neuroplasticity

**DOI:** 10.1007/s00018-018-2948-z

**Published:** 2018-10-22

**Authors:** Frank M. J. Sommerlandt, Axel Brockmann, Wolfgang Rössler, Johannes Spaethe

**Affiliations:** 1 Behavioral Physiology and Sociobiology (Zoology II), Biozentrum, University of Würzburg, Am Hubland, 97074 Würzburg, Germany; 2 National Centre for Biological Sciences, Tata Institute of Fundamental Research, Bellary Road, Bangalore, 560065 India

**Keywords:** Activity-regulated genes, Mapping tool, Honey bee, Long-term memory formation, egr-1, c-jun

## Abstract

Social insects show complex behaviors and master cognitive tasks. The underlying neuronal mechanisms, however, are in most cases only poorly understood due to challenges in monitoring brain activity in freely moving animals. Immediate early genes (IEGs) that get rapidly and transiently expressed following neuronal stimulation provide a powerful tool for detecting behavior-related neuronal activity in vertebrates. In social insects, like honey bees, and in insects in general, this approach is not yet routinely established, even though these genes are highly conserved. First studies revealed a vast potential of using IEGs as neuronal activity markers to analyze the localization, function, and plasticity of neuronal circuits underlying complex social behaviors. We summarize the current knowledge on IEGs in social insects and provide ideas for future research directions.

## Introduction: complex behaviors with miniature brains

Social insects like termites, ants, wasps, and bees build large colonies ranging from dozens to hundreds of thousands of individuals with overlapping generations and division of labor [1]. The multiplicity of tasks a colony is faced with is not coordinated by a centralized control system, but is rather exercised via self-organisation. Single individuals make decisions based on locally available information and interact with nestmates to produce a highly structured collective behavior [2, 3]. Even though their brains are rather small and comprise a neuronal network of relatively low complexity, social insects show sophisticated capabilities in terms of communication, navigation, and cognitive tasks. Paper wasps (*Polistes fuscatus*), for example, identify and learn individual faces of nestmates [4], *Cataglyphis* desert ants show complex navigational behaviors [5], and leaf-cutting ants (*Acromyrmex ambiguus*) learn to avoid fungus–noxious plants [6]. Moreover, social bees are capable of cognitive behaviors almost comparable to vertebrates [7–12]. Bumble bees, for example, show observational learning and cultural transmission of complex behaviors [13, 14], and honey bees are capable of time and place learning, communication of navigational information via dancing behavior [15–17], counting [18–20], and complex non-elemental forms of learning [21–23]. The richness in complex behaviors and the extensive collective interactions provide valuable opportunities to study underlying neuronal circuits, their plasticity, and processes involving memory formation (Table 1) and sets social insects apart from well-established genetic insect model organisms, such as *Drosophila,* or more simple invertebrate models like *Caenorhabditis.*

**Table 1:** Selected social insect models and examples of complex behaviors that show potential to study underlying neuronal circuits

Social insect model organism	Behavior of interest	References
Termites		
*Macrotermes natalensis*	Vibrational communication	Hager and Kirchner [24]
Ants		
*Cataglyphis spec.*	Navigation	Wehner [5]
*Ooceraea biroi*	Chemical communication	Trible et al. [25]
*Harpegnathos saltator*	Social stress and reproduction	Yan et al. [26]
*Camponotus floridanus*	Caste-specific polyethism	Zube and Rössler [27], Bonasio et al. [28]
Wasps		
*Polistes fuscatus*	Individual face recognition	Tibbetts [29]
Bees		
*Bombus terrestris*	Color learning	Lichtenstein et al. [30]
	Social learning/cultural transmission	Alem et al. [13]
*Bombus impatiens*	Route learning (traplining)	Saleh and Chittka [31]
	Decision making	Riveros and Gronenberg [32]
*Apis mellifera*	Dance communication	von Frisch [17]
	Time–place memory	Koltermann [16]
	Age-related polyethism	Withers et al. [33]
	Associative learning and memory	Giurfa [21]
	Age-related (neuro-) plasticity	Groh et al. [34]
*Apis florea*	Dance behavior	Dyer [35]

In combination with behavioral assays, several tools, including live (calcium) imaging, as well as pharmacological, electrophysiological, genetic, and histological approaches, have been developed to search for a memory trace in social insects. Studies on the processes of memory formation showed that brain plasticity is reflected in changes in the firing rate of neurons, alterations in their molecular and epigenetic profile, and in reorganization of the synaptic network [6, 36–42]. Particularly, the latter can be considered as the neuronal substrate of long-term memories (LTM) and behavioral plasticity [43]. However, the mechanisms providing the important link between transient changes of physiological properties of individual neurons and long-lasting structural reorganization or re-wiring of brain circuits are largely unexplored. A noted element of this transition process is the activation of a genomic cascade, which is precisely tuned and includes the expression of genes involved in neuronal physiology [44–46]. This leads, for example, to changes in the storage and mobilization of synaptic neurotransmitter-releasing vesicles and cell adhesion molecules (CAMs), which are essential for neuronal circuit formation (for a comprehensive review on molecular mechanisms involved in synaptic plasticity see Ho et al. [47]).

**Fig. 1:**
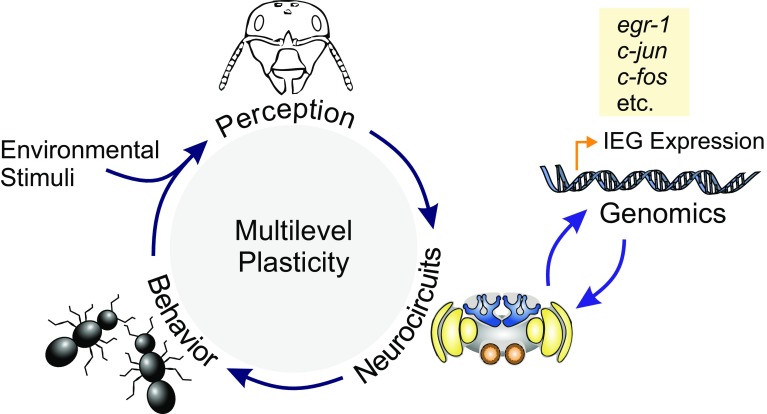
Social insects show extensive collective interactions and a striking plasticity in their behaviors. Stimuli from the environment and from interactions with other individuals are integrated and processed within neurocircuits by each colony member. Sensory exposure and learning activate a genomic response cascade in neurons that leads to changes in the structure and/or physiology of the neurocircuits. The first transcriptional wave after neuronal activation includes the expression of immediate early genes (IEG), which orchestrate plasticity at the neuronal, behavioral, and perceptual level. Their central role in controlling mechanisms of plasticity and the transient nature of their translation-independent expression makes IEGs promising markers for activated neuronal circuits

A unique group of genes that is expressed in the first transcriptional wave after neuronal activation are the immediate early genes (IEG). IEGs largely encode for transcription factors that orchestrate cellular homeostasis and neuronal plasticity. In vertebrates, IEGs are known to respond to neuronal stimulation in a rapid and transient fashion without the need of de novo protein synthesis [48–50]. Due to their transient expression that can peak within tens of minutes after stimulation, IEGs can be used as molecular markers in the search for neuronal circuits that contribute to the transition from short-term neuronal activation to long-lasting structural changes at the synaptic and neuronal network level. In social insects, this approach has not yet been established for routine use, although it would allow the study of elaborate behaviors in freely moving animals in the social context and under natural conditions (Fig. 1) [51–54]. Monitoring behavior-related IEG expression, therefore, is a very promising tool to access brain functions related to social behavior, sensory exposure and learning. It bears the potential to provide a highly attractive extension to already established neurobiological methods, like electrophysiological recordings, calcium imaging, and immunohistological approaches to analyze protein expression profiles (Table 2). A particular benefit of IEG expression analyses is that entire brains can be screened for neuronal activity, whereas other methods require a certain degree of prior knowledge on neuron populations and neuronal circuits that might be involved in the response to the applied stimulation paradigm. Therefore, the analysis of IEG activation may be particularly beneficial in identifying the brain regions or even neurons involved in complex behavioral processes like individual decision making, behavioral transitions, navigation, cognition, and advanced social communication.

**Table 2:** Comparison of advantages and limitations of different approaches for measuring neuronal activation and plasticity

	Genomic tools: immediate early genes	Electrophysiology/live (calcium) imaging	Circuit analyses/neuroanatomy: neuronal/synaptic connectivity
Investigating complex behaviors in freely moving animals	Yes	Limited (partially using implanted electrodes/objectives)	Yes
Investigating Pavlovian conditioning in harnessed animals	Yes	Yes	Yes
Accessing the brain in vivo	No	Yes	Very limited
Temporal resolution	Snapshot	Live image	Snapshot
Screening the complete brain for neuronal activity	Yes	No	Limited (requires quantitative screening for changes in synaptic circuits/neuropil volumes)

With the present review, we aim to provide an overview of the current knowledge on the use of IEGs as neuronal activity markers in social insects, particularly in the honey bee, and to discuss potential perspectives for a broader implementation in social insects.

## Molecular mechanisms of neuronal plasticity

**Fig. 2:**
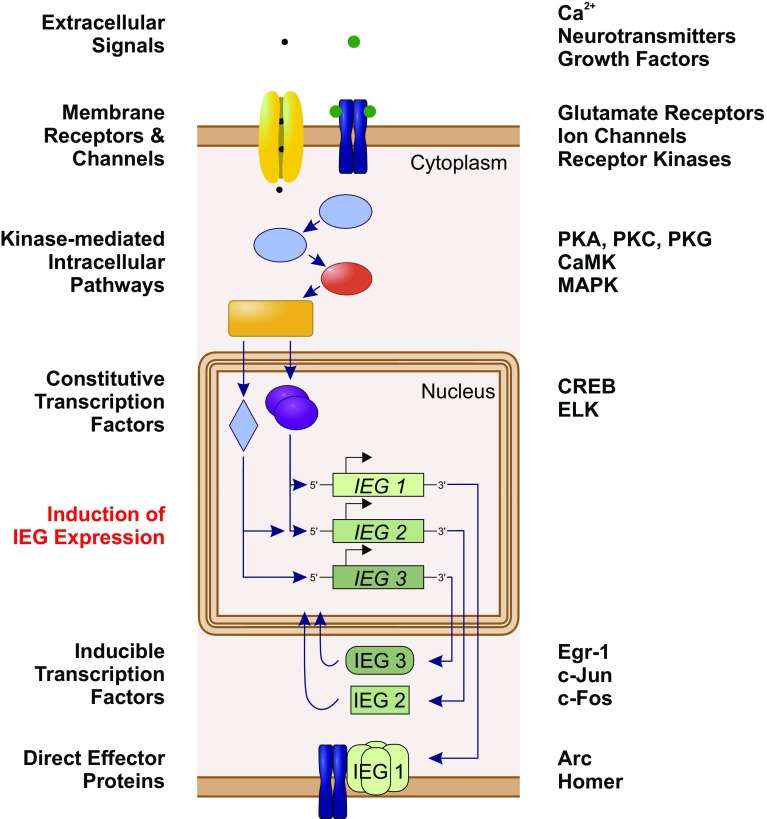
Intracellular activation cascade of immediate early genes (IEGs; left column) and examples of involved molecules and molecule classes (right column). Extracellular signals activate via membrane receptors and channels a series of intracellular biochemical pathways. Kinases then mediate the activation of constitutively expressed transcription factors that initiate the expression of IEGs. Protein products of IEGs can act either as inducible transcription factors to orchestrate the expression of downstream genes, or as direct effector proteins with implications in cell physiology and signaling

Social insect brains undergo plastic changes in the course of ontogenetic development and in response to sensory exposure, (pheromone) communication, as well as learning and memory processes. This is reflected in a modified neuropil structure, synaptic connectivity, firing properties of single neurons, and gene expression [46, 55–59]. In the context of memory formation, different phases can be distinguished that contribute to neuronal plasticity based on underlying molecular processes (Fig. 2). The first cellular responses to stimulation occur within seconds to minutes and include the activation of voltage-dependent Ca^2+^ channels or membrane receptors that respond to extracellular signals such as neurotransmitters and growth factors. This activation triggers a series of intracellular second messenger pathways that include phosphatases and protein kinases, e.g., protein kinase A (PKA) and Ca^2+^/calmodulin-dependent protein kinase II (CaMKII). Kinases then modify ion channels and constitutive transcription factors (transcription factors that do not necessarily require an activation but are rather permanently expressed) to orchestrate delayed neuronal responses [60–64].

Delayed responses last between hours and days and may result in permanent changes in neuronal properties and rearrangements of synaptic networks. On the molecular level, activation of constitutive transcription factors, e.g., the cAMP response element binding protein (CREB), leads to the expression of IEGs. This process constitutes the “first genomic response” to stimulation [44, 48, 49]. In analogy to the classical electrophysiological action potential (eAP), this “genomic action potential” (gAP; terminology introduced by Clayton [48]) represents a neuronal integration process which involves regulation of nuclear gene expression instead of membrane-associated ion channels. In contrast to the immediate synaptic transmission initiated by the eAP, the gAP regulates slower acting functional and structural modulations of the synaptic network via a pulse of increased transcription of IEGs. Activation of IEGs represents the first wave of gene transcription in response to neuronal stimulation and their expression is a prerequisite for transcription-dependent long-term neuronal plasticity. The expression of IEGs also occurs in the presence of protein synthesis inhibitors [65–67] and each IEG responds in a characteristic manner in distinct brain regions to different types or qualities of stimulation [68]. In the absence of sensory stimulation, most IEGs are expressed at low levels, with only few exceptions [69].

Protein products of IEGs are involved in a multitude of cellular processes with diverse functions that are important in the reorganization of neuronal networks [49]. In general, two classes of IEGs can be distinguished, based on the functional role of the encoded products. The first class encodes for proteins with direct implications in cell structure and signal transduction. These IEGs are directly involved in processes such as receptor modulation, vesicle storage, or synaptic trafficking and are, therefore, called direct effectors (e.g., *arc* and *homer1a*). The second class, comprising most of the commonly studied IEGs, encodes for inducible transcription factors (e.g., *egr*–*1*, *c*-*jun*, *c*-*fos*) which regulate the expression of downstream late-response genes involved in neuronal physiology [48, 66, 69] (Fig. 2). In both cases, transient cellular stimulation gets converted into long-term changes via the activation of a molecular response cascade.

The rapid and transient nature of their induction makes IEGs ideal markers for neuronal activation and their study offers two benefits: on the one hand, it helps understanding the molecular processes leading to modifications in synaptic functioning. On the other hand, as their expression indicates sites of neuronal activation, analyses of IEG activation patterns may provide important insights into the functional construction of the brain. In this way, the spatial distribution and temporal succession of activated neuronal circuits that are involved in the formation and storage of memories can be localized and analyzed.

## IEGs in studies of complex behaviors in vertebrates

Eukaryotic IEGs were first described in vertebrates, for which they are now routinely used in functional mapping studies to monitor neuronal activation [65, 66]. For most IEGs, peak mRNA levels are detectable around 30–60 min after stimulation onset and highest protein levels occur between 60 and 120 min after stimulation [70–73]. IEG induction in neurons was first demonstrated in response to seizures [74]. Since then, a vast number of studies reported functional links between the induction of IEG expression and social stimuli or complex behaviors, and perceptual stimulations associated with memory formation (reviewed in Refs. [48, 51, 65]). For example, IEG activation occurs in the hippocampus of rodents after visual, olfactory and spatial learning, and in the cortex when exploring novel environments [72, 75, 76]. In songbirds, IEG expression in the brain is induced when individuals are exposed to a novel conspecific song for the first time. After a song and its context became familiar by repetition, that particular song no longer induced the genomic response [70, 71, 77]. In addition, stimulus-enriched environments and drugs of abuse are known to activate IEG responses in specific brain parts [78, 79]. Dysregulated IEG expression was linked to the pathophysiology of human neurodegenerative disorders such as Alzheimer’s dementia and amyotrophic lateral sclerosis [80, 81], demonstrating their central role in orchestrating neuronal plasticity.

Besides investigating the function of IEGs within neuronal systems, IEGs were also used to monitor activation of neuron populations in co-expression experiments. The simultaneous detection of activity-regulated IEGs and cell markers such as neurotransmitters and receptors helped to identify neuron populations involved in complex vertebrate behaviors, such as mating and aggression [82] or social stress [83].

## The honey bee: insect model for monitoring IEG expression

To shed light on the molecular and neuronal processes involved in complex learning and memory formation in a social context, the honey bee became an important and fruitful insect model system (reviewed in Refs. [42, 64, 84, 85]). Accelerated by the sequencing of the honey bee genome, molecular tools have been developed to study intracellular pathways in neurons and to determine the role of behaviorally relevant genes [86, 87].

So far, IEG expression patterns in honey bees were rarely analyzed at the protein level (to our knowledge, only one study analyzed IEG protein levels in the context of ontogenetic development; [88]), whereas most studies analyzed mRNA levels using RT-qPCR and in situ hybridization, respectively (Table 3). Activation of IEGs or genes regulated by them were compared between different pupal and adult stages [89, 90], and between individuals performing different tasks like nursing the brood, dancing to communicate a novel food source to nestmates, and foraging for nectar or pollen [91, 92]. Behavioral approaches aiming to stimulate IEG expression in honey bees included more general stimulation like seizures induced by awakening from anesthesia [92–95], exposure to light [96] or plant and pheromonal odors [96–98], and sucrose feeding (food reward stimulation; [99]). In addition, more specific behaviors were correlated with IEG expression, for example, feeding of sucrose or pollen of different qualities [100], as well as different aspects of orientation flights [92, 95, 101] and foraging activity [102, 103].

**Table 3:** Main candidate IEGs investigated in honey bees

Gene	Stimulant	Effector sites	Method	References
*Amegr* (*Egr*-*1, zenk, zif/268, Krox*-*24, Stripe*)	Environmental novelty	MB ↑	mRNA: in situ hybridization, RT-qPCR	Lutz and Robinson [101]
	Seizure induction	AL ↑, OL ↑, MB ↑	mRNA: in situ hybridization, RT-qPCR	Ugajin et al. [94]
	Ontogenetic development: early to mid pupal stage	OL ↑	mRNA: in situ hybridization, RT-qPCR (isoform-specific)	Ugajin et al. [89]
	IPA or light	No effect	mRNA: RT-qPCR	Sommerlandt et al. [96]
	Foraging	Entire brain ↑	mRNA: RT-qPCR	Singh et al. [102]
	Time-dependent foraging	AL ↑, OL ↑, KC ↑	mRNA: in situ hybridization, RT-qPCR	Shah et al. [103]
	Nurse-forager-transition	Entire head	CAGEscan (Cap Analysis of Gene Expression: promotor region characterization of activated genes)	Khamis et al. [91]
*Amjra* (*c*-*jun*)	IPA	AL ↑	mRNA: RT-qPCR	Alaux and Robinson [97]
	IPA	AL (inconsistent effects)	mRNA: RT-qPCR	Alaux et al. [98]
	Sucrose feeding	AMMC ↑, MB ↑, LP ↑, GNGl ↑, OL ↑	mRNA: in situ hybridization, RT-qPCR	McNeill and Robinson [99]
	(a) Food type (b) Food value	(a) LP, AL, OL, MB (b) AMMC, AL, OL, MB, GNGl	mRNA: in situ hybridization	McNeill et al. [100]
	IPA or light	AL ↑, OL ↑, MB ↑	mRNA: RT-qPCR	Sommerlandt et al. [96]
*c*-*Fos* (*kayak*)	Ontogenetic development: embryonic, nymphal and adult stage	AL, MB	Protein: immunohistochemistry, immunocytochemistry, immunoblotting	Fonta et al. [88]
	Drone development	Mucus gland ↑	mRNA: RT-qPCR; cDNA Representational Difference Analysis (RDA)	Colonello-Frattini et al. [143]
	Bacterial infection	Fat body↑, oenocytes ↑	mRNA: RT-qPCR; whole genome microarray	Richard et al. [142]
	Exposure to xenobiotics	Not specified	mRNA: RT-qPCR	Cizelj et al. [90]
*hr38* (*Nr4a*)	Caste and division of labor	MB ↑	mRNA: in situ hybridization, RT-qPCR	Yamazaki et al. [145]
	Foraging	Entire brain ↑	mRNA: RT-qPCR	Singh et al. [102]
*kakusei*	Seizure induction, dancer vs. forager vs. nurse, reorientation	sKC ↑, OL ↑, AL ↑	mRNA: in situ hybridization, RT-qPCR	Kiya et al. [92]
	Seizure induction	OL, MB, DL ↑	mRNA: in situ hybridization, RT-PCR	Kiya et al. [93]
	(a) Seizure induction and thermal stimulation (b) IPA induction	(a) KC ↑ (b) No effect	mRNA: in situ hybridization, RT-qPCR	Ugajin et al. [146]
	Seizure induction, foraging, reorientation, light	OL ↑ AL (no effect)	mRNA: double-in situ hybridization, RT-qPCR	Kiya and Kubo [147]

↑ upregulation, *AL* antennal lobes, *AMMC* antennal mechanosensory and motor center, *DL* dorsal lobe, *GNGl* lateral gnathal ganglia (formerly termed as lateral suboesophageal ganglion), *IPA* isopentyl acetate, *KC* Kenyon cells, *LP* lateral protocerebrum, *MB* mushroom bodies, *OL* optic lobes, *sKC* small KC

## IEG candidates in honey bees: putative functions and pathways

Studies in honey bees focused on five candidate IEGs. Four of these genes (*egr*-*1*, *c*-*fos, Hr38,* and *c*-*jun*) have well studied orthologs in vertebrates and encode for regulators of gene transcription. Among metazoans, these transcription factors show a high degree of conservation in the structure of their functional domains and, presumably, in involved upstream and downstream regulatory networks [89, 94, 104–107]. However, in addition to the above-mentioned transcription factors, one candidate IEG in honey bees encodes for a non-coding RNA called *kakusei* that might be specific to honey bees [93]. We discuss the candidate genes in more detail below.

### egr (zif-268, zenk, stripe, ngfi-a, krox-24; Fig. 3a)

One of the best studied IEGs both in vertebrates and in the invertebrate *Aplysia* is *egr*-*1* [108, 109]. This gene encodes a transcription factor belonging to the early growth response (Egr) protein family. The family comprises four members (Egr-1 to Egr-4) that are expressed in various isoforms [68]. A common structural feature of all members is a highly conserved DNA-binding domain comprising three tandem Cys_2_His_2_ zinc finger motifs, that target a GC-rich sequence of nine consecutive nucleotides (5′-GCG C/GGG GCG-3′), termed Egr-binding sequence (EBS; [68, 110–112]). EBS can be found in the promotors of several genes involved in the ecdysteroid-signaling pathway [91] and nerve cell functioning, including genes encoding synapsin I and II [113, 114], and acetylcholinesterase [115]. In addition to motifs responsive for CREB and Elk–1 transcription factors [116], *Egr* genes also contain the EBS motif, resulting in a negative feedback loop [117]. All Egr protein members target the same DNA consensus sequence, yet their activity is regulated by interactions of a variable peptide sequence outside the DNA-binding domain with other proteins or co-factors [118].

**Fig. 3:**
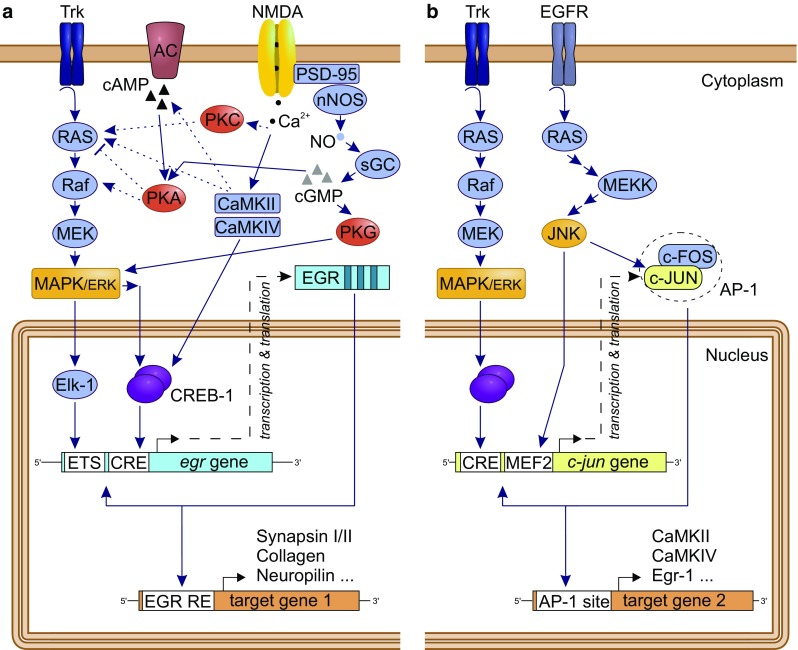
Potential cellular pathways and major players for the induction of the immediate early genes *egr* (**a**) and *c-jun* (**b**), and downstream targets, with focus on pathways previously linked to learning and memory in honey bees [42, 64, 85, 158]. **a** Activation of tyrosine receptor kinases (Trk) by neurotrophins induces via Ras (G protein) and Raf (kinase) the MAPK/ERK pathway, resulting in an activation of the transcription factors (TF) Elk-1 and/or CREB-1. By binding to their consensus target sequences (ETS and CRE sites), the TFs induce the transcription of *egr*. The Egr protein product in turn functions as a TF and activates the transcription of various late-response target genes. A list of candidate downstream genes in honey bees can be found in Khamis et al. [91]. Egr additionally auto-regulates its own expression by interacting with the promotor of the *egr* gene. Alternative regulation pathways include the cAMP-PKA signaling pathway and NMDA receptor-mediated activation of PKC or CaM kinases. **b** Activation of *c*-*jun* is also mediated by the MAP/ERK pathway. Another MAPK signaling pathway includes the c-jun NH2-terminal kinase (JNK), which activates *c*-*jun* expression by binding of the MEF2 site in the promotor. c-Jun protein is regulated through phosphorylation by JNK and forms homo- or heterodimers (e.g., with c-Fos) resulting in the activator protein 1 (AP-1) complex, which regulates gene transcription via AP-1 binding sites on the DNA. c-Jun also auto-regulates its own transcription. Pathways compiled after [64, 112, 113, 135, 180–182]

*Egr* genes were first discovered in a screening assay searching for factors determining the differentiation of embryonic rat neuroblasts into neuron-like cells [119]. Besides its activation by the neuropeptide NGF in neuroblasts, *egr* expression is also induced by a variety of pharmacological and physiological stimulants, including glutamate and NMDA, dopamine and cocaine, caffeine, ethanol, visual and tactile stimulation, restrainment, and learning (reviewed in [68]). The putative role of Egr in learning and memory formation is of increasing interest in vertebrate neuroscience. Cerebral expression of Egr family members is induced by various learning tasks including visual associative learning (macaques [120]), spatial learning (rats [75], mice [121]), vocal communication and auditory memory formation (zebra finches [70]), as well as the formation of olfactory long-term memories (mice [122]). In all cases, the formation of new associations is required for the activation of *egr* genes, as sensory stimulation and motor responses alone are not sufficient to increase expression levels. Members of the Egr family are critically involved in long-term potentiation (LTP) processes, for which the activation of *egr* genes is required for the maintenance of late phases of LTP and the formation of LTM (reviewed in Refs. [112, 122, 123]). The degree to which Egr is up-regulated after learning correlates with the persistence of LTP [124].

In honey bees, only a single orthologous *egr* gene (named *Amegr* in *Apis mellifera*) is known, located on chromosome 15 and expressed in three distinct isoforms of unknown function [89, 96]. Induction of *Amegr* mRNA expression was observed in the developing brain [89], after awakening from CO_2_ anesthesia [94, 95], in mushroom bodies after orientation flights in young foragers [95, 101], and in response to conspecific intruders [125]. In addition, foraging bees had, on average, higher *Amegr* levels compared to nursing bees [91], and foragers showed an increase in *Amegr* levels when starting to continuously visit a feeding site [102]. In contrast, exposure to isolated stimuli like a pulse of light or alarm pheromone was not sufficient to induce *Amegr* expression in harnessed bees [96]. By analyzing the promoter regions of differentially expressed genes between nurses and foragers, Khamis et al. [91] identified 424 genes that are potentially regulated by the Amegr protein. This underlines the wide range of functional connections of this transcription factor. So far, no direct role of *Amegr* expression in learning and memory processes was shown, even though its implication in orientation [101], foraging [91, 102, 103], and drone mating flights [126] strongly suggest such a function. Singh et al. [102] showed that a foraging-dependent upregulation of *Amegr* is associated with an activation of downstream genes involved in learning and memory. Another open question is whether the three expressed isoforms of *Amegr* have different functions or show brain-neuropil-specific expression patterns.

### c-jun (jra) and c-fos (kayak): formation of the dimeric AP-1 transcription factor complex (Fig. 3b)

The activator protein-1 (AP-1) transcription factor is composed of homo- or heterodimers formed between Jun and Fos protein family members. Both, c-Jun and c-Fos, belong to bZIP-type DNA-binding transcription factors, which are characterized by a basic DNA-binding domain and the “Leucine zipper” dimerization domain [127, 128]. AP-1 regulates genes by binding to the DNA consensus sequence 5′-TGA G/C TCA-3′, which is present in the promotor region of target genes and called TPA responsive element (TRE) or AP-1 site (reviewed in [129, 130]). AP-1 regulates genes involved in neuronal signal transmission.

C-Jun is a highly conserved member of the Jun family, which is encoded by an intronless gene that is expressed in a single isoform, both in vertebrates and honey bees [96, 131]. The mRNA consists of one of the longest 5′ untranslated regions known, possibly indicative of a strong posttranscriptional regulation, which is in accordance with the pronounced differences between *c*-*jun* mRNA and protein levels found in stimulated cells [132, 133]. The expression of the *c*-*jun* gene is regulated by constitutively expressed transcription factors such as CREB and ATF, in response to various stimuli including growth factors, cytokines, and UV radiation [134]. In addition, *c*-*jun* is positively autoregulated by AP-1, resulting in signal amplification and signal prolongation [133, 135]. Jun proteins include a Jun domain, which can be modified by posttranslational phosphorylation, e.g., by c-Jun N-terminal kinases (JNK; [136, 137]). In honey bees, the *c*-*jun* gene (known as *Apis mellifera* Jun-related antigen, *Amjra*) was shown to be expressed in cell somata throughout the honey bee brain [99]. Expression of *Amjra* was induced in the antennal lobes (AL) after stimulation with isopentyl acetate, a component of the bees’ alarm pheromone [96–98], with plant odors [97], and after light exposure [96]. In the lateral protocerebrum, mushroom bodies (MB), and optical lobes (OL), *Amjra* expression was increased after sucrose feeding [99, 100]. Interestingly, the response of *Amjra* after stimulation seems to be globally in the entire brain and independent of the stimulus modality [96, 99].

*c*-*fos*, in turn, is expressed in two different isoforms in *Drosophila* and, presumably, in honey bees [132, 138]. *Fos* transcription is mediated by CREB, and in contrast to *c*-*jun*, *c*-*fos* is downregulated by its own protein product and the AP-1 complex [134, 139–141]. Studies in human cells revealed that while both genes get rapidly and transiently induced, high *c*-*jun* mRNA levels last considerably longer than *c*-*fos* levels [129]. In honey bees, c-Fos protein levels were increased during development and in antennal-lobe somata of adult bees [88]. Regulation of *c*-*fos* mRNA expression was analyzed for honey bees mostly in the context of immunoreaction and pesticide exposure [90, 142], as well as in mucus gland of differentially aged drones [143]. To our knowledge, no analysis of brain mRNA expression of *c*–*fos* has been done so far.

### Hr38 (Nr4a)

The hormone receptor 38 (Hr38) in insects bears structural homology to the vertebrate nuclear receptor related 1 protein (NURR1, also known as NR4A). It is regulated by Egr and has been suggested to fulfil important neuronal functions by mediating ecdysteroid signaling [91, 102]. Expression of *hr38* was used to monitor neuronal activation in *Drosophila* and moths (*Bombyx mori*) [53, 144]. In honey bees, foragers possess elevated *hr38* expression as compared to nurses and queens [145]. Only recently was *Hr38* expression shown to be induced following seizure and orientation flights [95], during foraging behavior [102] and in the context of aggression [125]. The *hr38* gene is likely expressed in more than one isoform.

### Other potential IEG candidates in honey bees

The non-coding nuclear RNA *kakusei* was found to be induced in the densely packed inner compact Kenyon cells of the mushroom bodies by a variety of stimuli including seizure following anesthesia, during the behavioral transition from nurses to forager bees, and after reorientation in foragers [92, 93]. Even though its function is unclear, one inducible and several constitutively expressed transcript variants were identified [93]. Additional IEG candidates were discovered by seizure induction experiments and included genes encoding protein kinases and nucleotidyltransferases [95]. However, *kakusei* does not appear to have any orthologous genes in other taxa, and for the other two gene groups orthologous genes are still awaiting annotation. Therefore, these genes might be currently less suitable for (comparative) functional IEG studies in social insects.

## Outlook and future directions

Several studies in recent years emphasized the potential of IEGs as genomic markers for neuronal activation in social insect brains [92, 100–103]. This approach helped, for example, to identify brain regions in honey bees that are involved in the evaluation of food type and value [100] or that are active during orientation flights [92, 101] and foraging [102, 103]. Honey bees showed an increased IEG expression even in anticipation of foraging behavior, particularly in the small Kenyon cells of the mushroom bodies [103]. Kiya and Kubo [147] went one step further and demonstrated a behavior-dependent IEG activation of biochemically identified neuron populations in the optic lobes by simultaneously measuring expression of *kakusei* and the neurotransmitter *gamma*-Aminobutyric acid (GABA) in a double-in situ hybridization assay. This approach is particularly promising as in insects most neuronal cell bodies are located in the cell body rind surrounding the neuropil mass and often cannot be associated with a specific brain region (except for the mushroom bodies). Double labeling could, therefore, help to identify neuron types and neuronal circuits based on biochemical markers.

Promising brain neuropils to study the neuron-specific expression and differential activation of IEGs in more detail are the insect mushroom bodies (MB) and the central complex (CX). MBs are brain centers for multimodal sensory integration and learning and memory, and functional correlations between the connectivity of MB synaptic microcircuits and various behaviors were found in bees [36, 148], wasps [149, 150], and ants [6, 151]. Depending on the type of stimulation, properties of the synaptic network in MBs can change: for example, sensory exposure leads to presynaptic pruning and postsynaptic sprouting [56, 152], and associative learning and long-term memory formation is correlated with presynaptic sprouting [6, 36]. The CX is involved in sensory integration and high-order motor control and was shown to express neuronal plasticity induced by complex visual learning and memory formation [151, 153–155]. The specific programs underlying plasticity in both neuropils are likely orchestrated by different sets of IEGs or, alternatively, the same IEGs expressed in different sets of neurons [103, 156]. Therefore, IEG-based approaches are applicable at two different levels: first, identification of relevant IEGs, followed by double-in situ hybridization could help to identify the type of neuron populations that are involved in the different physiological programs and types of neuroplasticity. Second, inhibition of the expression of particular IEGs should, for example, impact the level of synaptic connectivity and result in a reduced memory capacity [157]. To test the latter, IEG knockdown assays, like in vivo RNA interference (RNAi), combined with behavioral learning experiments or neuroanatomical analyses are promising. Such an approach, for example, uncovered the importance and the distribution of the activated “memory protein” CaMKII, which has a dual function both as activator and target of IEGs in long-term memory formation [158–160].

IEG studies are also promising for the identification of neurocircuits involved in processing sensory information. The use of a magnetic compass, for example, is known from various animals like birds, mammals, crustaceans, and also social insects such as ants and honey bees [161–164]. Despite the broad distribution of magnetoreception in the animal kingdom, the sensory pathways and perceptual mechanisms are mostly unexplored. In insects, a sensory mechanism and putative brain areas responsible for processing magnetic information are completely unknown, making the use of electrophysiological recording or live-imaging techniques inefficient. Screening for a magnetic-field driven induction of IEGs, for example, during learning or orientation excursions in naïve animals, might be a promising approach to identify involved neurocircuits. A similar approach could help to uncover neurobiological mechanisms that underlie the honey bee’s dance communication [165]. Between dancing bee species and species that lack the ritualized dances (e.g., bumble bees), no apparent differences were found in sensory projections [166]. Adaptations in the neuronal circuitry that facilitate the specific dance behavior thus seem to be rather small and a comparative IEG expression analysis might help to identify such differences.

As IEG expression is likely in many cases highly specific regarding the stimulation paradigm and the behavioral responses [144], a systematic analysis of the role of different stimulation programs and contexts is required to specify the functional role of candidate genes. In songbirds, for example, expression of an *egr* homolog is significantly increased in the brain when birds hear a song of their own species, as compared to heterospecific songs, and decreases when the song has been made familiar by repetition [70, 71]. Therefore, known IEGs in social insects need to be tested in a larger variety of developmental stages, stimulus repetition rates or behavioral contexts as IEG expression can be highly selective for one or all of these parameters. Honey bees and bumble bees, for example, respond to the same scent marks deposited by conspecifics at food sources either by avoiding or approaching them, depending on previous foraging success on marked flowers [167]. In leaf-cutting ants of the genus *Acromyrmex*, the degree of allogrooming behavior as a part of the social immune response is regulated depending on previous infections of the colony [168]. Such adaptive and context-specific behaviors are potentially mediated by a differential regulation of IEGs in inhibitory or excitatory neuronal circuits. Therefore, a careful dissection of the relationship between stimulation properties and the characteristics of IEG activation is essential. In addition, one has to keep in mind that neuronal activation might occur without the induction of IEGs or that the expression of IEGs might occur independently from neuronal stimulation [169]. For example, an isolated exposure of honey bees to olfactory or visual stimuli does not induce *egr* expression, even though neuronal activation in this paradigm is indicated by the expression of the IEG *jra* [96].

To study the potential of IEG-based approaches, Pavlovian conditioning under harnessed conditions as it is now established in various bee [30, 170–173] and ant species [174, 175] is a promising complement to experiments with free-moving animals. In such an approach, stimulus features can be gradually dissected when individuals are kept under controlled conditions [176, 177] and the brain can be accessed in vivo [38, 178]. Approaches monitoring IEG expression thus provide a unique possibility to analyze the neuronal control of naturally motivated behaviors both in natural (social) environments and under more isolated and controlled laboratory conditions.

Finally, while putative IEGs are now available for honey bees, other social insect species need to be screened for homologous genes, to broaden the field of IEG applications and the understanding of gene functions. For example, IEG-based comparative studies among different social insect species could help to unveil the neuronal correlates that facilitate the emergence of sociality. In contrast to vertebrates (social brain hypothesis), the level of sociality in insects is not reflected in simple correlations with brain (neuropil) volumes [179]. Therefore, an alternative approach to reveal general neuronal constraints underlying social systems might be the IEG-based detection of neuronal circuits involved in social tasks, the processing of social signals, such as recruitment pheromones or cuticular hydrocarbons, and the regulation of behavioral plasticity. In this context, *egr*-*1* might be particularly useful, as it was shown to respond to social stimuli in different vertebrate species [49, 51].

The hitherto success and obvious benefits of IEG analyses in vertebrates and pioneering studies in honey bees should encourage more researchers in behavioral neuroscience to pursue this new approach. We, therefore, aim to advance the usage of this promising tool to other social insect species, as comparative studies are needed to uncover the mechanisms underlying their sophisticated behaviors in the social context.
